# Application of CRISPR/Cas13a system on the rapid detection of *Salmonella* spp.

**DOI:** 10.1371/journal.pntd.0014150

**Published:** 2026-03-23

**Authors:** Yongxin Huang, Wenli Liang, Mingyao Huang, Yingying Deng, Zhenyi Huang, Changhan Ai, Weiqing Tan, Lingxiao Jiang

**Affiliations:** 1 Department of Laboratory Medicine, Zhujiang Hospital, Southern Medical University, Guangzhou, Guangdong, China; 2 Guangdong Provincial Clinical Research Center for Laboratory Medicine, Guangzhou, Guangdong, China; 3 School of Mechanical and Aerospace Engineering, Nanyang Technological University, SingaporeSingapore; Yale University School of Medicine, UNITED STATES OF AMERICA

## Abstract

**Background:**

*Salmonella* spp. infections can manifest in various clinical symptoms, from asymptomatic carriage to gastroenteritis, and even severe sepsis. Given the rapid progression of the disease and its potential to cause severe outcomes or trigger cluster outbreaks, making the detection of *Salmonella* spp. critically important. Although broth enrichment culture is considered the gold standard, it is time-consuming and involves multiple steps, making it difficult to meet urgent diagnostic needs. Hence, prompt and precise detection of *Salmonella* spp. is crucial not only for early diagnosis and effective treatment, but also for preventing transmission, controlling outbreaks, and screening asymptomatic *Salmonella* carrier.

**Methods:**

This study developed a clustered regularly interspaced short palindromic repeats (CRISPR)/CRISPR associated (Cas) -SE assay that integrated the advantages of the recombinase polymerase amplification (RPA) and CRISPR/Cas13a system for detecting *Salmonella* spp. The clinical performance of CRISPR/Cas13a-SE assay was evaluated by a cohort of 94 inpatients with diarrhea and three prospective studies.

**Results:**

The CRISPR/Cas13a-SE assay can be completed within 60 minutes, and its limit of detection was 100 fg/μL. Compared to the broth enrichment culture, the CRISPR/Cas13a-SE assay demonstrated a sensitivity of 87.5% and a specificity of 98.8% in a cohort of 94 inpatients with diarrhea. In our prospective studies involved three distinct cohorts: 1,662 food handlers, 211 outpatients with diarrhea, and 154 inpatients with Gram-negative bacteremia. Compared with broth enrichment culture, CRISPR/Cas13a-SE assay had a high concordance rate of 98.79% (1,642/1,662), 99.52% (210/211), and 100.00% (154/154) respectively.

**Conclusions:**

We demonstrated that the CRISPR/Cas13a-SE system showed excellent detection performance for infectious diarrhea caused by *Salmonella* spp. The combined use of CRISPR/Cas13a-SE with the blood culture method enhances the rapid diagnosis of invasive salmonellosis, which is crucial for early target-based therapy. Additionally, screening of asymptomatic *Salmonella* carrier will be benefit for disease prevention and control.

## Author summary

Prompt and precise detection of *Salmonella* spp. is crucial not only for early diagnosis and effective treatment, but also for preventing transmission, controlling outbreaks, and screening asymptomatic *Salmonella* carrier. In this study, we established a novel CRISPR-based method for *Salmonella* spp. rapid diagnosis. Our findings indicate that CRISPR/Cas13a-SE is more sensitive, particularly in cases of low bacterial load, and is simpler and faster than the PCR method. Its potential for cost-effective development, simplicity, and convenience suggests that CRISPR/Cas13a-SE could be developed into a low-cost point-of-care testing (POCT) device, utilizing basic fluorescence-detecting equipment to make the process faster and more accessible.

## Introduction

*Salmonella* spp., a member of the Enterobacteriaceae family, is responsible for causing salmonellosis. *Salmonella* spp. infection can cause symptoms like asymptomatic infection, gastroenteritis, and even sepsis after invading the body [1]. Vulnerable populations, including infants, the elderly, and immunocompromised individuals, are at higher risk of life-threatening complications like bacteremia and sepsis and in some cases, meningitis or osteomyelitis. Diagnosing *Salmonella* spp. infections is challenging due to their non-specific clinical features, diverse manifestations. For patients suspected of *Salmonella* infection, the broth enrichment culture method is considered the gold standard for detection. However, this multi-step method typically takes 2–3 days to yield results.

Additionally, asymptomatic carriers of *Salmonella* spp. are potential sources of clustered outbreaks. Persistent infection in these carriers constitutes an independent risk factor for abdominal aortic aneurysm and may also contribute to the development of inflammatory bowel disease or gastrointestinal cancer [2–4].

Nonetheless, the broth enrichment method is clearly unsuitable for large-scale screening of asymptomatic carriers. Therefore, there is an urgent need for a rapid, user-friendly, and highly sensitive method for both the diagnosis of *Salmonella* spp. and the screening of asymptomatic carriers.

Although PCR-based molecular diagnostic methods offer faster turnaround times, it is mainly employed in large tertiary hospitals and remains impractical for use in community healthcare settings.

The CRISPR (clustered regularly interspaced short palindromic repeats)-Cas (CRISPR-associated) systems have emerged as the most widely used genome editing technology worldwide [5]. Based on the activated collateral cleavage activity of Cas13a, the CRISPR/Cas13a system has been developed into an effective tool for molecular diagnostics [6,7].

To address the unmet clinical needs for rapid and accurate detection of *Salmonella* spp., we have developed a rapid, user-friendly, and highly sensitive method for the identification of *Salmonella* spp., leveraging the precision of the CRISPR-Cas13a system, known as CRISPR/Cas13a-SE. We conducted a cohort of 94 inpatients with diarrhea and three prospective studies to evaluate its diagnostic performance. Our results demonstrate that CRISPR/Cas13a-SE exhibits excellent potential for clinical pathogen detection, enabling timely identification of *Salmonella* spp. for improved clinical decision-making. Furthermore, this system effectively identifies asymptomatic carriers, offering substantial value for public health by helping reduce Salmonella transmission and prevent associated complications.

## Materials and methods

### Ethics statement

This study was reviewed and approved by the Zhujiang Hospital Ethics Committee Review Board (2019-KY-029–01). All participants provided electronically signed informed consent for specimens to be stored and used in this research.

### Sample collection and detection

Fecal specimens were collected from 94 diarrhea inpatients at Zhujiang Hospital in Guangzhou, China from August 2019 to May 2021.

Additionally, three prospective studies were conducted from January 2022 to December 2023, including fecal samples from 1,662 food handlers, 211 outpatients with diarrhea, as well as blood culture broths from inpatients with Gram-negative bacteremia.

### Cas13a protein

The Cas13a purification procedures were described previously [8,9]. First, the open reading frame (ORF) of LwCas13a was synthesized and cloned into the pET-28a expression vector. The resulting plasmid, encoding a His-tagged Cas13a protein, was transformed into *Escherichia coli* BL21 (DE3) competent cells. Following bacteria expansion, isopropyl-β-d-thiogalactopyranoside (IPTG) was added to a final concentration of 1 mM to induce protein expression at 20 °C for 15 hours. After induction, bacterial cells were collected and lysed to obtain the clarified supernatant containing soluble His-tagged Cas13a. Protein was purification through Ni-affinity chromatography, the final purified protein was aliquoted and stored at –80 °C. Protein purity and yield were evaluated by SDS-PAGE and Western blot analysis.

### Fecal culture

For the direct culture method, fecal specimens were directly inoculated onto the Xylose-Lysine-Deoxycholate (XLD) agar plates (Guangzhou Detgerm Microbiogical Science Ltd.) and incubated at 35 °C, 18–24 hours. Presumptive *Salmonella* spp. colony was selected for identification via matrix-assisted laser desorption/ionization-time of flight mass spectrometry (MALDI-TOF MS, Autof ms 1000, Autobio).

For the broth enrichment culture method, fecal specimens were firstly enriched in SALMONELLA-SHIGELLA broth (SS broth, Guangzhou Detgerm Microbiogical Science Ltd.) at 35 °C, 16–18 hours. Then subcultured on the XLD agar plates at 35°C overnight. Presumptive *Salmonella* spp. colony was selected for identification via MALDI-TOF MS.

### Bacteria strains

*Salmonella typhimurium*, *Enterobacter cloacae*, *Klebsiella pneumoniae*, *Citrobacter braakii*, *Escherichia coli*, *Pseudomonas aeruginosa*, *Acinetobacter baumannii*, *Enterococcus faecalis* and *Staphylococcus aureus* were collected in the Department of laboratory of Zhujiang Hospital. The DNA was extracted by the Bacterial Genomic DNA Extraction Kit, and the DNA concentrations were measured by the Qubit dsDNA HS Assay Kit (Q32851, Invitrogen).

### Blood culture

Positive blood culture broths were subculture on blood agar plates (Guangzhou Detgerm Microbiogical Science Ltd.) at 35 °C, 16–24 hours. The isolated colony was selected for identification via MALDI-TOF MS.

### DNA rapid extraction

1.5 mL of saline mixed fecal specimen or broth culture samples were centrifuged at 2,000 g for 3 minutes, after which the supernatant was collected to a new tube and centrifuged at 16,000 g for 5 minutes. The sediment was mixed by vortex and heated at 100 °C for 10 minutes. Following another centrifugation at 16,000 g for 5 minutes, the supernatant containing DNA was used as the template for CRISPR/Cas13a-SE detection. Specifically, the total hands-on time required for the operator is approximately 5–7 minutes, as only brief manual actions such as adding reagents, mixing, and transferring the sample are needed. The remaining time consists of incubation or centrifugation steps, during which no manual intervention is required. The entire DNA extraction process takes approximately 30 minutes.

### Oligos and gRNA

Primers with an appended T7 promoter used in the recombinase polymerase amplification (RPA) for the invA gene (110 bp, NC_003197.2) amplification were listed below, forward primer 5'-TAAT ACGA CTCA CTAG AGGG TTCG CCAA TGGC GGCG AATT ACGA GCAG-3' and reverse primer 5'- GGGT CAAG GCTG AGGA AGGT ACTG CCAG AGGT C -3'. We used crRNA for Cas13 (5'- GGGG AUUU AGAC UACC CCAA AAAC GAAG GGGA CUAA AACU GCGA AUAA CAUC CUCA ACUU CAGC AGA -3') and ssRNA probe (5'-6-FAM-UUUUUC-BHQ-3') for CRISPR detection after RPA amplification.

### CRISPR/Cas13a-SE

**Fig 1:**
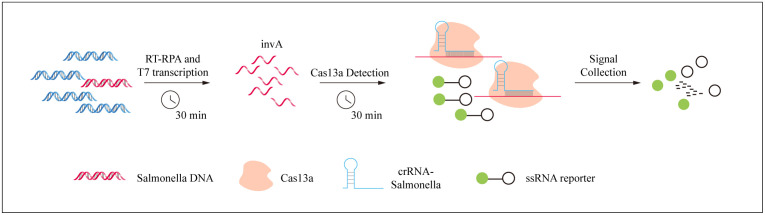
Schematic diagram of CRISPR/Cas13a-SE method for rapid *Salmonella* spp. detection. The CRISPR/Cas13a-SE detection system combines an RPA step, subsequent T7 transcription, and a Cas13 detection step. Briefly, RPA reactions were operated at 37°C for 30 minutes. The amplification product was then added to the CRISPR reaction mix and was incubated at 37°C, the fluorescence signals were monitored for 30 minutes.

The CRISPR/Cas13a-SE detection system combines an RPA step, subsequent T7 transcription, and a Cas13 detection step, as described previously [9]. Briefly, RPA reactions (25 μL total volume) containing 2.5 μL of template DNA, 0.5 μM of each primer, 1 × reaction buffer, 14 mM of magnesium acetate, and the RPA enzyme mix were incubated at 37°C for 30 minutes. The amplification product was then added to the CRISPR reaction mix, which consisted of 1 μL crRNA (33.3 nM), 1 μL Cas13a (66.7 nM), 1.25 μL of nucleotide triphosphate each (10 mM), 0.25 μL T7 RNA polymerase (New England Biolabs) and 0.5 μL of ssRNA reporter (166 nM). The final reaction mix was incubated at 37°C, and fluorescence signals were monitored using the ABI7500 (Thermo-Fisher Scientific) for 30 minutes. The total time required for the CRISPR/Cas13a testing procedure is about 60 minutes (Fig 1).

We calculated the fold changes relative to the no-template control to normalize the fluorescent signal. The formula of fluorescence fold change value (FC) for each sample was calculated as FC = (end-point fluorescence value of the sample)/ (BC end-point fluorescence value) [9]. A cutoff of 2.1 was set in this study.

### Evaluation of the limit of detection

To evaluate the limit of detection, the DNA of the *Salmonella* spp. strain (ATCC 14028) was purified. The concentration was determined using Qubit (Thermo-Fisher Scientific). A ten-fold serial dilution was performed with nuclease-free water to achieve the desired concentrations (1 fg/μL, 10 fg/μL, 100 fg/μL, 1 pg/μL, and 10 pg/μL) to evaluate the limit of detection (LOD). Ten replicate experiments were conducted to evaluate the lower detection limit of the CRISPR/Cas13a method, defined as the lowest template DNA concentration at which at least nine out of ten replicate experiments yielded positive results.

### Statistical analysis

As appropriate, a comparative analysis was performed using the Pearson χ2 test, Fisher exact test, or the Student t-test. Data analysis was conducted using GraphPad Prism 9. Statistical significance was determined with *P* values <0.05. All tests were two-tailed unless otherwise specified.

## Results

### Development of CRISPR/Cas13a-SE assay

We have engineered a CRISPR/Cas13a-SE detection system for *Salmonella* spp., integrating a recombinase polymerase amplification (RPA) reaction, T7 transcription, and a Cas13a detection step to achieve a rapid and highly sensitive diagnostic method. Initially, we designed primers and CRISPR RNA (crRNA) targeting the highly conserved invA gene, we screened multiple pairs of RPA primers and several CRISPR crRNAs targeting different regions within *Salmonella* spp. invA gene and finally chose the best primers. The *Salmonella* spp. DNA was extracted using a heating method. This strategy is easy to use without demanding complicated machines and materials. We optimized the reaction system and evaluated its sensitivity and specificity. Eventually, we establishing our CRISPR/Cas13a-SE assay for *Salmonella* spp. detection (Fig 1).

### Performance verification of CRISPR/Cas13a-SE

**Fig 2:**
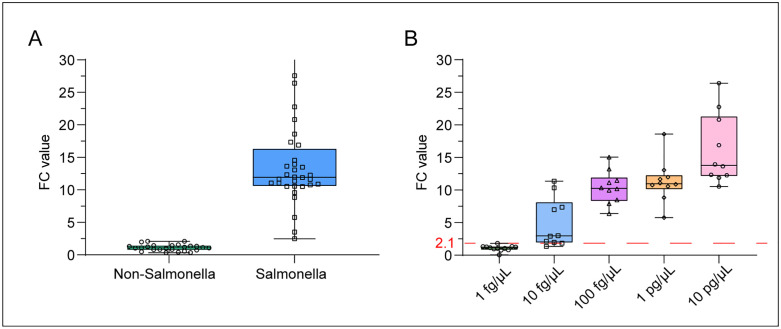
Analytical assessment of the specificity, sensitivity and limit of detection of CRISPR/Cas13a-SE. Evaluation was performed by testing various microbes (A) and different copy numbers of *Salmonella* spp. genomic DNA **(B)**.

To confirm specificity, we tested two human DNA samples and 23 non-*Salmonella* spp. strains, such as *Salmonella typhimurium*, *Enterobacter cloacae*, *Klebsiella pneumoniae*, *Citrobacter braakii*, *Escherichia coli*, *Pseudomonas aeruginosa*, *Acinetobacter baumannii*, *Enterococcus faecalis* and *Staphylococcus aureus*. None of these interference samples triggered a false positive reaction (Fig 2A).

To determine the analytical sensitivity of the CRISPR/Cas13a-SE assay, we tested 37 *Salmonella* spp. strains isolated from our laboratory, which include the top three serotypes in southern China: *Salmonella Stanley*, *Salmonella enteritidis* and *Salmonella typhimurium* [10]. The CRISPR/Cas13a-SE assay successfully detected all strains, with a fluorescence change (FC) above 2.1 (Fig 2A).

We further evaluated the limit of detection (LOD) of CRISPR/Cas13a-SE by serial dilutions of purified DNA of the *Salmonella typhimurium* strain (ATCC14028). As Fig 2B shows that at 10 fg/µL, the fluorescence signals varied across replicates and did not consistently exceed the threshold (8 of 10 replicates was detected as positive), which did not meet our predefined criteria for a reliable LOD. At 100 fg/µL, the fluorescence increase was clear, stable, and statistically significant, with all replicates showing positive detection. Therefore, although the assay begins to show detectable activity below 100 fg/µL, 100 fg/µL is the lowest concentration that satisfies all LOD performance criteria, and thus was chosen as the reported detection limit.

These analytical evaluations suggest that the CRISPR/Cas13a-SE assay has 100% sensitivity and specificity for detecting isolated bacterial colonies, making it a promising molecular assay for *Salmonella* spp. detection.

### Clinical diagnostic evaluation of CRISPR/Cas13a-SE

Following our analytical study, we assessed the diagnostic potential of the CRISPR/Cas13a-SE assay in a clinical setting. We enrolled 94 diarrhea inpatients in a cohort study and detected *Salmonella* spp. using four methods: broth enrichment culture, direct culture, CRISPR/Cas13a-SE, and CRISPR/Cas13a-SE after broth enrichment. For each CRISPR/Cas13a-SE assay run, we included a positive control of purified *Salmonella* spp. DNA and a blank control to ensure accurate results.

Compared to the gold standard, a 97.9% (92/94) concordance were observed in CRISPR/Cas13a-SE. The CRISPR/Cas13a-SE assay identified seven out of eight *Salmonella* spp.-positive samples and identified 85 culture-negative specimens correctly. The single undetected sample by CRISPR/Cas13a-SE was successfully detected after broth enrichment, suggesting a low concentration of *Salmonella* spp. might lead to a false-negative result.

Interestingly, one sample that tested negative by broth enrichment culture yielded a positive result with the CRISPR/Cas13a-SE assay. This sample was from a patient post-antibiotic treatment, suggesting that the bacteria may have been non-viable and thus undetectable by culture, but detectable by CRISPR/Cas13a-SE due to the presence of their DNA. This highlights the higher sensitivity of CRISPR/Cas13a-SE, albeit without the ability to distinguish between live and dead bacteria.

The CRISPR/Cas13a-SE assay showed a sensitivity of 87.5% (7/8), a specificity of 98.8% (85/86), a positive predictive value (PPV) of 87.5%, and a negative predictive value (NPV) of 98.8% (Table 1).

**Table 1:** Comparison of the clinical performance of the CRISPR/Cas13a-SE and culture methods.

Assay and result	Broth enrichment culture			% (95% CI)			
	Positive	Negative	Total	Sensitivity	Specificity	PPV	NPV
CRISPR/Cas13a-SE							
Positive	7	1	8	87.5 (52.9-99.4)	98.8 (93.7-99.9)	87.5 (52.9-99.4)	98.8 (93.7-99.9)
Negative	1	85	86				
Total	8	86	94				
CRISPR/Cas13a-SE after broth enrichment							
Positive	8	1	9	100.0 (67.6-100.0)	98.8 (93.7-99.9)	88.9 (56.5-99.4)	100.0 (95.7-100.0)
Negative	0	85	85				
Total	8	86	94				
Direct culture							
Positive	1	0	1	12.5 (0.6-47.1)	100.0 (95.7-100.0)	100.0 (5.1-100.0)	92.5 (85.3-96.3)
Negative	7	86	93				
Total	8	86	94				
Broth enrichment culture							
Positive	8	0	8	100.00 (67.6-100.0)	100.00 (95.7-100.0)	100.00 (67.6-100.0)	100.00 (95.7-100.0)
Negative	0	86	86				
Total	8	86	94				

Additionally, the CRISPR/Cas13a-SE assay, the CRISPR/Cas13a-SE after broth enrichment method and direct culture method showed a high concordance rate of 97.87% (92/94), 98.94% (93/94)., and 92.55% (87/94) respectively, all the methods show high concordance rates compared with the 'gold standard' method, however, when analyzing the sensitivity and specificity, it indicated that the direct culture method showed a high false negative rate, with an extremely low sensitivity of 12.5% (1/8). Our results showed that the CRISPR/Cas13a-SE assay not only showed a high concordance with the broth enrichment method, but also showed a high sensitivity and specificity.

Furthermore, the direct culture method had a significantly lower sensitivity of 12.5%, emphasizing the necessity of broth enrichment for accurate detection by the culture method. The CRISPR/Cas13a-SE assay offers a time-saving advantage of 18–24 hours in *Salmonella* spp. detection.

### Application evaluation of CRISPR/Cas13a-SE

**Fig 3:**
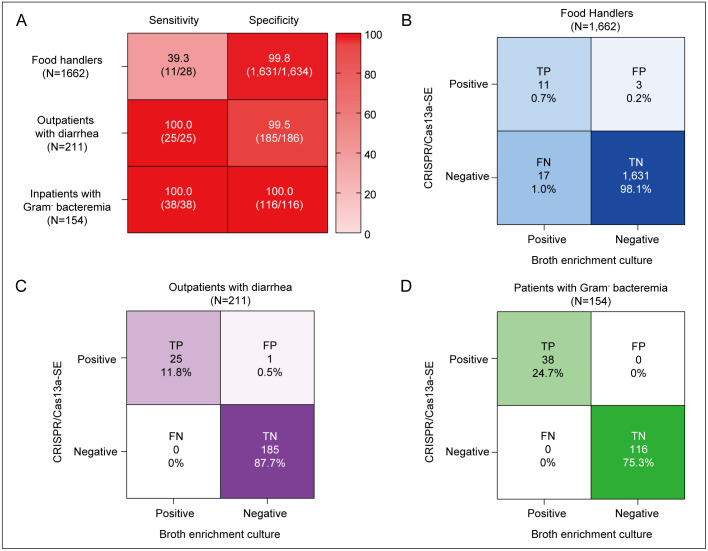
Sensitivity, specificity, and confusion matrix of the different methods and aspects. **(A)** Clinical Diagnostic Evaluation of CRISPR/Cas13a-SE in three prospective cohorts **(B–D)** The confusion matrix using the CRISPR/Cas13a-SE in food handlers, outpatients with diarrhea and patients with Gram-negative bacteremia respectively. The values are not normalized. TP: True positives, FP: False positives, FN: False negatives, TN: True negatives.

Recognizing the promising diagnostic performance of the CRISPR/Cas13a-SE assay, we conducted three prospective studies to evaluate the performance of CRSPR/Cas13a-SE for *Salmonella* spp. detection under different condition. These studies involved three distinct cohorts: 1,662 food handlers, 211 outpatients with diarrhea, and 154 inpatients with Gram-negative bacteremia. Each specimen was concurrently tested using both the conventional broth enrichment culture method and the CRISPR/Cas13a-SE assay (Fig 3A).

In the prospective cohort of 1,662 food handlers, compared with broth enrichment culture, CRISPR/Cas13a-SE assay had a high concordance rate of 98.79% (1,642/1,662, Fig 3B). This finding indicates that direct testing of fecal samples using CRISPR/Cas13a-SE is an effective method for screening *Salmonella* spp. carriers.

Among outpatients with diarrhea, the results from CRISPR/Cas13a-SE showed a remarkably high consistency with those from broth enrichment culture, at 99.5% (210/211), suggesting that CRISPR/Cas13a-SE can detect almost all *Salmonella* spp. in patients with diarrhea symptoms. Notably, only one sample tested positive by CRISPR/Cas13a-SE and negative by broth enrichment culture, as illustrated in Fig 3A and 3C. This sample was also from a patient post-antibiotic treatment, this discrepancy may be attributed to the higher sensitivity of CRISPR/Cas13a-SE, which surpasses that of the enriched culture method, though it lacks the capacity to differentiate between live and dead bacteria.

We further assessed the detection capability of CRISPR/Cas13a-SE in patients with bloodstream infection of *Salmonella* spp. Based on the Gram stain results from positive blood culture bottles, positive blood culture broths were subsequently subjected to testing with the CRISPR/Cas13a-SE method. Our findings revealed that the CRISPR/Cas13a-SE method produced results that were completely concordant with those of the routine workflow (as depicted in Fig 3A and 3D). The CRISPR/Cas13a-SE method demonstrated outstanding performance, achieving 100.0% sensitivity, specificity, positive predictive value (PPV), and negative predictive value (NPV). Moreover, it offers a turnaround time within 1.5 hours, in contrast to conventional culture-based methods that typically require 16–24 hours.

## Discussion

*Salmonella* spp. infections can manifest in various clinical symptoms, from asymptomatic carriage to gastroenteritis, and even severe sepsis. For patients suspected of *Salmonella* infection, the gold standard, the broth enrichment culture method is time-consuming and involves multiple steps. Asymptomatic *Salmonella* carriers can serve as a significant source of infection, posing a serious risk to public health. However, the broth enrichment method is clearly unsuitable for large-scale screening of asymptomatic carriers. Therefore, there is an urgent need for a rapid, user-friendly, and highly sensitive method for both the diagnosis of *Salmonella* spp. and the screening of asymptomatic carriers.

In China, broth enrichment culture is the most commonly used method for detecting *Salmonella* spp. [1]. However, this technique typically requires 2–3 days to yield results, limiting its suitability in urgent scenarios such as sepsis and public health emergencies.

Although PCR technology can be employed for the detection of *Salmonella* spp., the high site conditions and personnel qualification requirements limit the application and promotion of this technology. In China, its use is currently concentrated within Centers for Disease Control and Prevention (CDC) for the management of public health events. [11,12].

The CRISPR-Cas system, derived from bacterial adaptive immunity, has revolutionized genome editing and is now being harnessed for molecular diagnostics. Leveraging the activated collateral cleavage activity of Cas13a, the CRISPR/Cas13a system has been developed as an effective tool for molecular diagnostics. Zhang's lab developed the first CRISPR-Cas13a-based Specific High-Sensitivity Enzymatic Reporter UnLOCKing (SHERLOCK) detection method [13]. Since then, in particular, CRISPR/Cas13a system has demonstrated its potential in detecting a range of pathogens, from Zika to COVID-19, underscoring its versatility and effectiveness [14,15]. This underscores the CRISPR-Cas13a system's considerable potential for pathogens detection [16].

Our development of a CRISPR/Cas13a-based method (CRISPR/Cas13a-SE) for *Salmonella* spp. detection addresses the need for a rapid, user-friendly, and highly sensitive diagnostic tool. In our study involving 94 diarrhea inpatients, the detection rates were 8.51% using fecal broth-enrichment culture and 9.57% using CRISPR/Cas13a-SE with broth enrichment method, highlighting the method's high sensitivity and specificity, with a detection limit of 100 fg/μL.

We have developed a novel rapid, user-friendly, and highly sensitive method for the identification of *Salmonella* spp., leveraging the precision of the CRISPR-Cas13a system, known as CRISPR/Cas13a-SE. We conducted a cohort of 94 inpatients with diarrhea and three prospective studies to evaluate its diagnostic performance. Our findings demonstrate that CRISPR/Cas13a-SE is rapid and user-friendly, exhibiting high sensitivity and specificity, with a limit of detection as low as 100 fg/μL (approximately 20 copies/μL).

As shown in Table 1, statistical analysis revealed a significant difference in *Salmonella* spp. detection rates between direct culture (1/94, 1.1%) and broth-enriched culture (8/94, 8.5%), but there was no significant difference between CRISPR/Cas13a-SE without enrichment (8/94, 8.5%) and after broth-enrichment (9/94, 9.6%). This suggests that broth enrichment is necessary for culture method, while CRISPR/Cas13a-SE could achieves effective detection without it.

The CRISPR/Cas13a-SE method demonstrated superior sensitivity (7/8, 87.5%) and negative predictive value (85/86, 98.8%) compared to the direct culture method, which showed a sensitivity of only 12.5% (1/8). This indicates that CRISPR/Cas13a-SE can detect *Salmonella* spp. in stool samples more rapidly and sensitively than traditional culture methods, reducing both time and consumable costs, and is promising to improve clinical diagnosis and laboratory efficiency.

Blood culture is a standard diagnostic approach for identifying invasive *Salmonella* spp. infections. Upon receiving a positive report for blood culture samples, we immediately collected these samples for further analysis. Initial identification of the bacteria was performed using Gram staining, followed by cultivation on agar plates. Subsequently, we utilized matrix-assisted laser desorption/ionization-time of flight mass spectrometry (MALDI-TOF) for rapid bacterial identification, which traditionally requires up to 24 hours [17]. The combination of CRISPR/Cas13a-SE with conventional blood culture methods enhances the diagnosis of invasive salmonellosis, facilitating timely antibiotic treatment and preventing disease progression in patients with severe comorbidities. offering a substantial advantage for pathogen diagnosis, especially for patients with compromised immune systems.

Furthermore, CRISPR/Cas13a-SE aids in screening *Salmonella* spp. carriers, contributing to bacterial decolonization and reducing the risk of complications such as aortic aneurysms.

This study has several limitations that should be acknowledged. First, although the overall detection performance was robust, the number of positive clinical samples was relatively limited, which may limit the statistical power and generalizability of our findings. Additionally, some low-concentration samples near the detection threshold produced variable fluorescence signals, indicating a need for further optimization of reaction conditions to improve robustness at very low target levels. Finally, although the CRISPR/Cas13a-SE assay demonstrated high sensitivity and specificity, the assay performance was evaluated under controlled laboratory conditions, and further validation in diverse clinical settings with broader populations is required.

Our findings indicate that CRISPR/Cas13a-SE is more sensitive, particularly in cases of low bacterial load, and is simpler and faster than the PCR method. Its potential for cost-effective development, simplicity, and convenience suggests that CRISPR/Cas13a-SE could become a standard *Salmonella* spp. testing method. CRISPR/Cas13a-SE significantly reduced detection time and showed great potential in pathogens diagnosis. This system can assist clinical physicians by timely identification of pathogens, which is crucial for precise therapy to control disease progression and spread, especially in patients with invasive infections.

CRISPR/Cas biosensing systems can integrate simple visual signal readouts with quantitative and multiplexing capabilities. Therefore, CRISPR/Cas13a-SE could be developed into a low-cost point-of-care testing (POCT) device, utilizing basic fluorescence-detecting equipment to make the process faster and more accessible.
